# Self-Assembly of Protein Monolayers Engineered for Improved Monoclonal Immunoglobulin G Binding

**DOI:** 10.3390/ijms12085157

**Published:** 2011-08-15

**Authors:** Anton P. Le Brun, Deepan S. H. Shah, Dale Athey, Stephen A. Holt, Jeremy H. Lakey

**Affiliations:** 1 Institute for Cell and Molecular Biosciences, The Medical School, Newcastle University, Framlington Place, Newcastle upon Tyne, NE2 4HH, UK; E-Mail: abn@ansto.gov.au; 2 Orla Protein Technologies Ltd, Biosciences Centre, International Centre for Life, Times Square, Newcastle upon Tyne, NE1 4EP, UK; E-Mails: deepan.s.shah@orlaproteins.com; proteins@orlaproteins.com; 3 ISIS Neutron Facility, STFC Rutherford Appleton Laboratory, Harwell Science and Innovation Campus, Didcot, Oxfordshire, OX11 0QX, UK; E-Mail: sph@ansto.gov.au

**Keywords:** membrane protein, gold, immunoglobulin, self-assembled monolayer, surface plasmon resonance

## Abstract

Bacterial outer membrane proteins, along with a filling lipid molecule can be modified to form stable self-assembled monolayers on gold. The transmembrane domain of *Escherichia coli* outer membrane protein A has been engineered to create a scaffold protein to which functional motifs can be fused. In earlier work we described the assembly and structure of an antibody-binding array where the Z domain of *Staphylococcus aureus* protein A was fused to the scaffold protein. Whilst the binding of rabbit polyclonal immunoglobulin G (IgG) to the array is very strong, mouse monoclonal IgG dissociates from the array easily. This is a problem since many immunodiagnostic tests rely upon the use of mouse monoclonal antibodies. Here we describe a strategy to develop an antibody-binding array that will bind mouse monoclonal IgG with lowered dissociation from the array. A novel protein consisting of the scaffold protein fused to two pairs of Z domains separated by a long flexible linker was manufactured. Using surface plasmon resonance the self-assembly of the new protein on gold and the improved binding of mouse monoclonal IgG were demonstrated.

## 1. Introduction

Protein arrays are becoming increasingly important. They have uses in biosensors, tissue engineering and proteomics. There are many challenges in developing new protein arrays and one direction of research is the coupling of array technology with the well established and described deposition of self-assembling monolayers (SAM) on gold [1]. Simple organic molecules with a thiol functional group can be immobilised to gold *via* a gold-thiolate bond, providing for the facile preparation of well characterised stable orientated layers on smooth surfaces [2]. Peptides and proteins with thiol-containing cysteine residues in their amino acid sequence can be included into mixed SAM on gold creating functional protein arrays [3,4] and membrane proteins are especially suitable since they naturally assemble into layers [5]. Membrane proteins with a β-barrel structure are particularly well suited to immobilisation as they can be specifically immobilised to a gold surface with a controlled orientation, retain their structure and function once immobilised and can be engineered to perform different functions [6].

**Figure 1.**
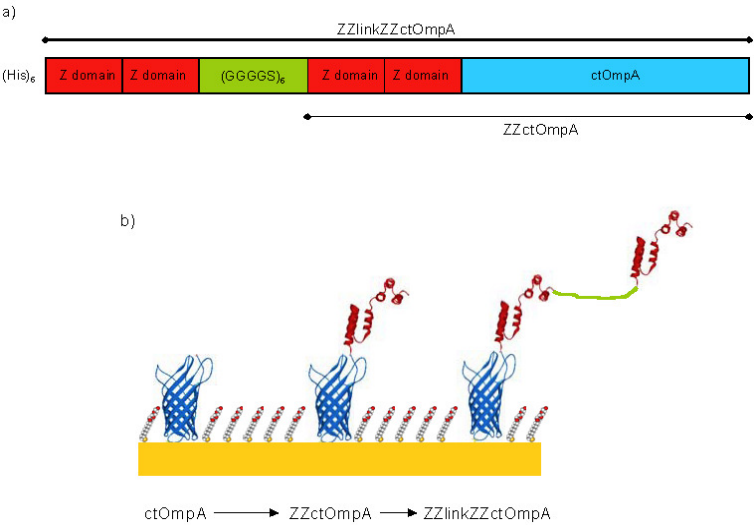
A schematic of ZZlinkZZctOmpA array protein. (**a**) A cartoon of the 5’ prime to 3’ prime sequence showing each part of the protein; and (**b**) A model of the array proteins on gold. The protein graphics were made in SWISS PDB viewer [21] using PDB files 1QJP for OmpA [8] and 1N2Q for the Z domains [22]. The ctOmpA part is in blue and the Z domains in red. The linker region is in green and is shown as unstructured. The filling molecule is also shown and is not a true representation of its packing.

Outer membrane protein A (OmpA) from *Escherichia coli* is a bacterial outer membrane protein with an N-terminal 8-stranded β-barrel transmembrane domain and a soluble *C*-terminal periplasmic binding domain [7,8]. The transmembrane domain of OmpA (tmOmpA) has formed the basis of a scaffold for inclusion in SAMs on gold. The tmOmpA has been engineered such that there is a cysteine residue for gold immobilisation in a periplasmic turn. Furthermore it has also been circularly permuted (ctOmpA) so that the N and C termini are exposed at the membrane surface allowing the addition of protein domains/motifs. Previously we have described fusing the Z domain of *Staphylococcus aureus* protein A to the *N*-terminus of ctOmpA (ZZctOmpA) [9]. The Z domain binds immunoglobulin G (IgG) at its constant region leaving the variable regions still free to bind antigen. The structure of ZZctOmpA in solution and surface immobilisation has been investigated [9,10]. Neutron reflectivity revealed that gold surface bound ZZctOmpA retained its structure when surrounded by a thioalkane filling molecule and that bound IgG was most likely to be in an upright conformation. Whilst an array of ZZctOmpA binds rabbit polyclonal IgG very tightly, mouse monoclonal IgG dissociates from the array more readily [10]. This is a problem because mouse monoclonals provide the specificity for a single epitope and are commonly used in immunodiagnostic assays as the primary capture antibody. In this paper we describe a novel strategy to reduce the dissociation of mouse monoclonal IgG2a from a membrane protein array and increase binding. We have shown that the stoichiometry of surface bound ZZctOmpA and IgG is one to one [10]. A single IgG molecule has two Z domain binding regions: one per heavy chain. To reduce the dissociation rate constant we aim to occupy both Z domain binding regions simultaneously using a linker system where a tandem pair of Z domains is linked by a flexible polypeptide linker to another Z domain pair which is then fused to ctOmpA (ZZlinkZZctOmpA) (Figure 1). The creation of the ZZlinkZZctOmpA can have two outcomes for IgG binding: either one IgG will bind per array protein as described above or the signal from the array will be increased due to an increased capacity for IgG binding from the simultaneous binding of two IgGs. The proposed polypeptide linker is a repeat of GGGGS. This type of linker was chosen as the small residues are less likely to interfere with other protein domains, it has a flexible random coil structure and the serine residue provides aqueous solubility [11]. Glycine-serine linkers have successfully been used by others to link antibody and hormone fragments [12,13] and for linking N and C termini of proteins in circular permutation [14].

## 2. Results and Discussion

### 2.1. Cloning

The gene encoding the ZZlinkZZctOmpA protein was constructed in stages. First a DNA segment encoding the linker sequence was synthesized by Epoch Biolabs (Sugar Land, Texas, USA) and supplied cloned into vector pBSK4D5F. Second, the linker sequence was PCR-amplified using primers Orla189F (5′-GATCCTCGAGCGGTGGGGGCGGTT-3′) and Orla190R (5′-GATCGTCGACTCAC CACCACCGCCTGAA-3′). The amplification was cloned into the *Xho* I site of plasmid pORLA76 [10], to create the linkXXctOmpA construct (named pORLA81). Finally, the region encoding a tandem pair of Z domains was PCR-amplified from plasmid pEZZ18 [15] using primers Orla1F (5′-GGGAGACCACAACGG-3′) and Orla166R (5′-CCATGTCGACGTGCTCGAATTCGCGTCTAC-3′) and cloned into the unique *Xho* I site of pORLA81 to create the gene encoding ZZlinkZZctOmpA (named pOrla82). At each stage, the clones with the desired insertion in the correct orientation were identified by analytical PCR, restriction digestion and gel electrophoresis. pORLA82 was verified by DNA sequencing.

### 2.2. Purification of Inclusion Bodies and Refolding

**Figure 2.**
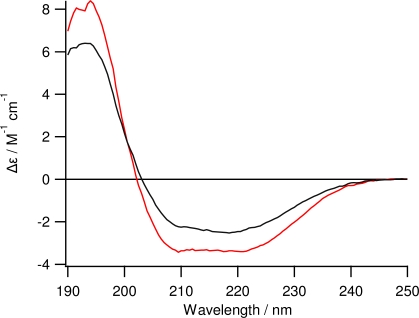
Circular dichroism spectroscopy of folded ZZlinkZZctOmpA (red trace) at 0.20 mg mL^−1^. The CD spectrum of ZZctOmpA (black trace) at 0.24 mg mL^−1^ is shown for comparison. Spectra were taken from 250 nm to 190 nm at a HT of less than 600 V using a 0.02 cm pathlength cuvette.

The ZZlinkZZctOmpA protein encoded on pORLA82 was expressed as inclusion bodies in *E. coli* BLR cells which have the *recA^-^* mutation that prevents recombination between stretches of identical DNA sequence such as the regions encoding the Z domains on pORLA82. The inclusion bodies were purified in 8 M urea first by immobilised metal affinity, and then by anion exchange, chromatography. A concentrated sample of protein in urea was slowly diluted into refold buffer with stirring and left to refold for 96 hours at 37 °C. The refolding was confirmed by band shifts on SDS-PAGE (not shown) and CD spectroscopy (Figure 2). The CD data clearly shows that there is more α-helix structure in ZZlinkZZctOmpA and that the protein is also folded. At over 50 kDa this is a comparatively large protein and with a flexible linker. Previous linkers have only used a single tandem repeat of gly-ser or a triple repeat of GGGGS. The ZZlinkZZctOmpA has a sextuple repeat of GGGGS, which, to our knowledge is the largest repeat of this type of linker successfully cloned, purified and refolded into a functional structure for an engineered protein. Long peptide linkers contain proline residues in their sequence which, due to having no amide hydrogen to form hydrogen bonds, suppress secondary structure [16]. However the presence of proline is undesirable in this case as it would add a structural rigidity that would impinge on IgG binding. Charged residues are also left out of the linker structure to avoid unwanted interactions between the linker and the IgG molecule.

### 2.3. Self-Assembly on Gold

**Figure 3.**
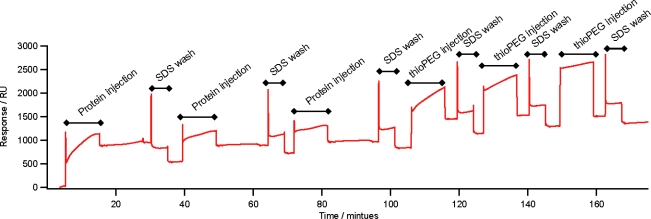
SPR trace of the assembly of ZZlinkZZctOmpA on gold. The assembly was carried out on a BIAcore X instrument using Au sensor chips. 25 μL of ZZlinkZZctOmpA (0.20 mg mL^−1^) was injected in to the flow cell at 5 μL min^−1^. Each protein injection was followed with an injection of 1% SDS at 5 μL min^−1^ to remove any non-specifically bound protein. After protein depositions a filling molecule, thioPEG was deposited. ThioPEG at 0.25 mg mL^−1^ in a solution of 1% OG 10 mM Tris-HCl pH 8.0 and 1 mM TCEP was injected into the flow cell at 5 μL min^−1^. As with the protein depositions each injection was followed by a 1% SDS wash to remove non-specifically bound material. The unlabelled sections of the trace are a PBS buffer wash.

ZZlinkZZctOmpA can assemble onto gold *via* the thiol group of the single cysteine residue in one of the periplasmic turns of ctOmpA. The surface assembly was measured using surface plasmon resonance (SPR). Three depositions of ZZlinkZZctOmpA were carried out leading to a total increase of 995 Response Units (RU) which equates to 1.18 × 10^10^ molecules mm^−2^ deposited onto the gold surface (Figure 3) (assuming 1 RU equates to 1 pg mm^−2^ of protein immobilised to the gold surface [17]). A 1% (w/v) SDS wash was used between each deposition to remove non-specifically bound protein. The total response equates to a surface coverage of 9.6%. The remaining surface was covered with a filling molecule which was a lipid-mimic, 1-mercaptoundec-11-yltriethylene glycol (thioPEG). The polyethyl glycol head group of the filling is resistant to non-specific protein adsorption [18] ensuring that any subsequent protein additions to the array will only bind *via* ZZlinkZZctOmpA. The assembly of the array is similar to that previously observed for protein immobilisation on gold. The surface coverage achieved is of the same magnitude as that reported for the engineered *E. coli* porin OmpF E183C [5] as well as ctOmpA and ZZctOmpA [10]. Removal of protein from the surface by the addition of thioPEG is unlikely due to the strong gold-thiolate bond between the surface and the thiol group of the cysteine residue. Significant protein removal has not been previously observed for other membrane proteins on gold by neutron reflection [10,19].

### 2.4. Antibody Binding

**Figure 4.**
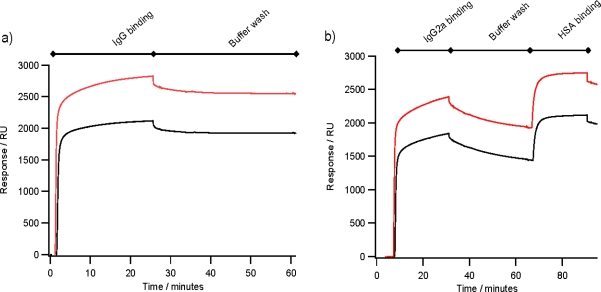
Comparing antibody binding between arrays of ZZctOmpA (black traces) and ZZlinkZZctOmpA (red traces). The arrays were assembled as in Figure 2 and IgG at 30 μg mL^−1^ in PBS was flowed over the arrays at 2 μL min^−1^. After the antibody injection PBS running buffer was flowed over the array to measure the dissociation of antibody from the array. 0.1 mg mL^−1^ HSA was injected at 2 μL min^−1^. (**a**) The binding of rabbit polyclonal IgG; and (**b**) Mouse monoclonal anti-HSA IgG2a binding.

Figure 4(a) shows typical binding of 30 μg mL^−1^ rabbit polyclonal IgG to an array of ZZlinkZZctOmpA with 2580 RU of bound IgG in this case, which is a similar level of binding to an array ZZctOmpA with 1955 RU. With both arrays there is very little dissociation of rabbit polyclonal IgG during the PBS wash. The ZZlinkZZctOmpA array also bound mouse monoclonal IgG2a (Figure 4(b)) with a total of 2258 RU bound at the end of the antibody injection. The bound mouse monoclonal IgG still retained the ability to bind antigen, which in this case was human serum albumin (HSA). A total of 676 RU of HSA was bound to the array in Figure 4(b), which corresponds to 33% of the available antigen binding sites being filled. The lack of saturation of the bound monoclonal IgG could be due to the dissociation of the IgG from the array during the antigen association.

**Figure 5.**
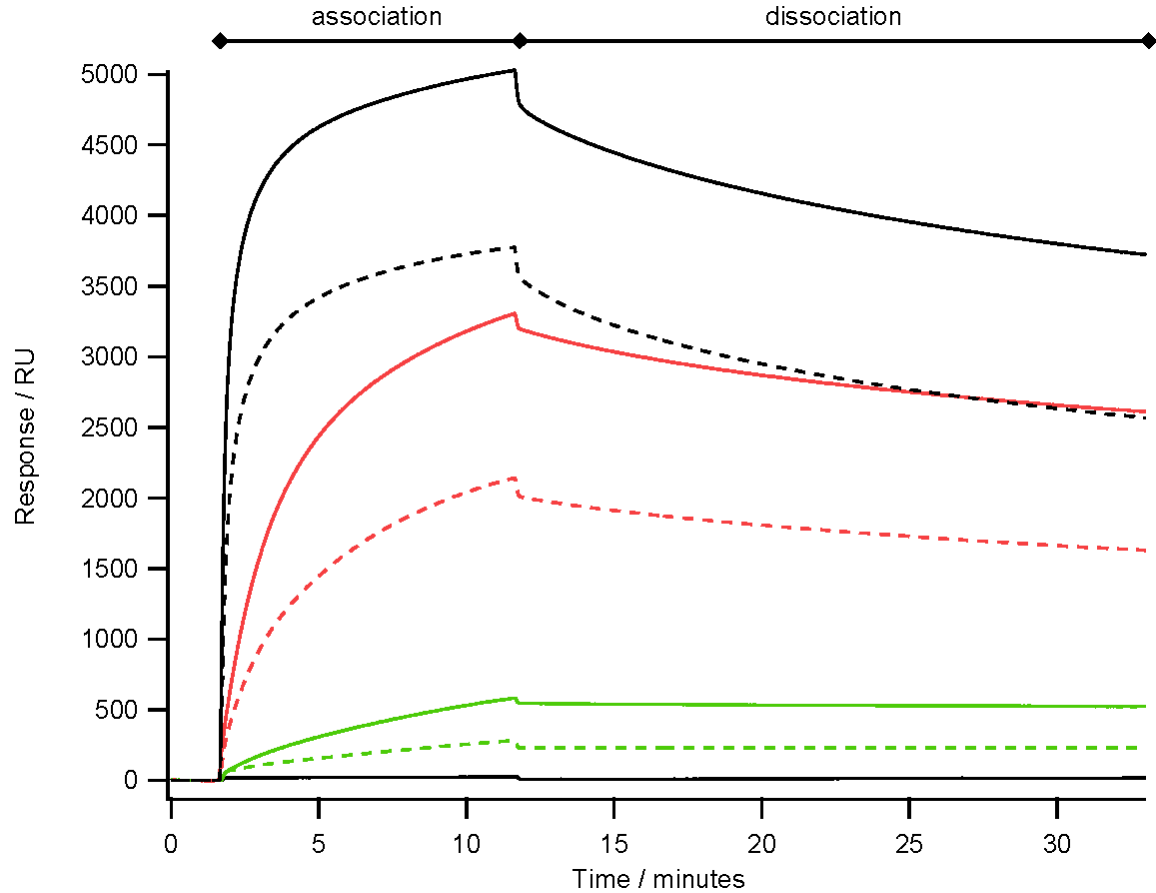
The binding of mouse monoclonal IgG2a to an array of ZZlinkZZctOmpA (solid traces) and ZZctOmpA (dashed traces) over a range of antibody concentrations. The antibody concentrations used were 480 nM (black), 40 nM (red) and 5 nM (green). A PBS only injection is also shown with 0 RU response. The antibody in PBS was flowed over the array at 10 μL min^−1^ and had a total contact time of 10 min before a dissociation time of 20 min. The array was regenerated with 100 mM glycine pH 2.0 between each antibody injection.

Previously, using an array of ZZctOmpA mouse monoclonal IgG2a dissociated from the array more readily than rabbit polyclonal IgG [10]. By analysing the binding of a range of concentrations of mouse monoclonal IgG2a over an array of ZZctOmpA and ZZlinkZZctOmpA it appears that the dissociation rate of antibody from the two arrays is similar (Figure 5). However the binding capacity of the ZZlinkZZctOmpA array is larger. This could be due to increasing the stoichiometry between the array protein and antibody i.e. two IgG molecules are binding to one ZZlinkZZctOmpA molecule.

An explanation for this may come from small angle X-ray scattering (SAXS) of blue fluorescent protein and green fluorescent protein linked by either 3× or 4× GGGGS which showed that the linker mainly adopted a compact structure and that this structure could have been driven by protein-protein interactions between the two fluorescent proteins [20]. The SAXS data also showed that the linkers were flexible enough to reach a maximum extension of 120 Å. Assuming an average amino acid length in a polypeptide chain of 3.5 Å then this would mean that the length of our GGGGS sextuplet linker would be 135 Å (length of linker plus one Z domain) which would be enough to span the Fc fragment of the IgG molecule and still allow for steric hindrances and van der Waals interactions. However if the linker is adopting a range of conformations from compact to extended then this may drive an increased capacity for multiple IgG binding rather than simultaneous binding of both Z domain binding regions on a single IgG.

## 3. Experimental Details

### 3.1. Materials

All chemical reagents were purchased from Sigma-Aldrich (Poole, Dorset, UK) or Melford Laboratories (Ipswich, Suffolk, UK) unless otherwise stated. Materials for molecular biology were from Promega or Fermentas. Columns for protein purification and gold surfaces for SPR were from GE Healthcare.

### 3.2. Molecular Biology

Gene amplification was by polymerase chain reaction (PCR). The PCR mix consisted of 2.5 μL of 10× reaction buffer, 1.0 μL of the forward and reverse primers at 100 μM concentration each, 2.5 μL of 2.5 mM deoxynucleotide triphosphates, 10 ng of template DNA, 0.5 μL of Fermentas DNA polymerase and water to make the volume up to 25 μL. The PCR was hot started at 95 °C for 30 seconds followed by 25 cycles of 95 °C for 30 seconds, 55 °C for 30 seconds and 68 °C for 4 min. The PCR products were assessed for yield and purity on a 1% (w/v) agarose gel stained with 0.05% ethidium bromide. The PCR products were purified and digested with restriction endonucleases using 1 unit of enzyme and relevant buffer. The plasmid vectors were linearised by cleavage with the *Xho* I enzyme and gel purified on a 1% agarose gel. Ligations were set up using molar ratios of 1:1, 1:3, 1:5 and 1:0 of vector to insert. The ligations used the T4 DNA ligase and were left to incubate at 4 °C overnight. The ligation products were then transformed into chemically competent *E. coli* XL1-blue cells and colonies picked and assessed for the presence of insert.

### 3.3. Protein Expression, Purification and Refolding

The ZZlinkZZctOmpA protein was expressed in *E. coli* BLR cells cultured at 37 °C in shaking flasks. Once the cells had reached an OD_600_ of ∼0.6 protein expression was induced by the addition of IPTG at a final concentration of 1 mM. The cells were harvested once they had reached the stationary phase of growth by centrifugation at 8000× *g* for 15 min and 4 °C. The cells were disrupted by incubation overnight at −20 °C in a detergent solution of Bugbuster (Novagen, Nottingham, UK) (using 10 mL for every gram of wet cell pellet) which was supplemented with DNase, RNase and lysozyme. The inclusion bodies were isolated by centrifugation at 12,000× *g* for 20 min and washed three times by homogenisation in a 1 in 10 dilution of Bugbuster in deionised water followed by centrifugation at 12,000× *g* for 20 min. The inclusion bodies were then solubilised in 8 M urea, 20 mM sodium phosphate pH 7.6, 500 mM NaCl and 20 mM imidazole. The proteins were column purified using an ÄKTA Prime FPLC system firstly by immobilised metal affinity chromatography (IMAC) using a HisTrap FF 1 mL nickel (II) ion column with elution typically in 15 mL of 250 mM imidazole, 8 M urea, 20 mM sodium phosphate pH 7.6 and 500 mM NaCl. The His_6_-tagged protein containing fractions were pooled, concentrated to a volume of 2.5 mL and then buffer-exchanged into 8 M urea, 30 mM ethanolamine pH 9.5 and 1 mM DTT using a PD10 column. The proteins were further purified using a 1 mL HiTrap Sepharose Q FF column using a 0 to 1 M NaCl gradient with elution of the protein typically at 120 mM NaCl. Pure protein fractions were concentrated to a volume of 1 mL using a Vivaspin 10,000 molecular weight cut-off spin concentrator. The proteins were refolded by slowly diluting the protein plus urea into refold buffer (20 mM Tris-HCl pH 8.0, 1% (w/v) n-octyl-β-*D*-glucopyranoside (OG), 0.1 mM EDTA and 1 mM DTT) by 1 in 10 and left to incubate for 72 hs at 37 °C to allow for complete refolding. Residual urea was removed by buffer exchange using a PD10 column equilibrated with refold buffer with 1 mM TCEP replacing DTT.

### 3.4. Circular Dichroism

Circular Dichroism spectroscopy was carried out using 0.02 cm pathlength demountable cuvettes in a Jasco J-810 spectropolarimeter. The protein concentration was determined by protein absorbance at 280 nm and circular dichroism was scanned from 250 nm to 185 nm ten times, averaged and a buffer blank was subtracted. The circular dichroism was expressed as Δε (M^−1^ cm^−1^).

### 3.5. Surface Plasmon Resonance

Au sensor chips were used for SPR experiments which have a bare gold surface deposited onto an optical slide. The gold surface of the chip was cleaned by incubation for 15 min in an acid piranha solution (70% (v/v) concentrated sulphuric acid 30% (v/v) hydrogen peroxide). The surface was then cleaned with 1% (v/v) Hellmanex (Hellma UK, Southend on Sea, Essex, UK) and rinsed with deionised water. The chip surface was passivated by 1% (v/v) BME solution in ethanol for 10 min followed by washing with 1% (w/v) SDS and distilled water.

The filling molecule, 1-mercaptoundec-11-yltriethylene glycol (thioPEG) (Prochmia Surfaces, Sopot, Poland) was diluted in a solution of 1% (w/v) OG, 20 mM Tris-HCl pH 8.0 and 1 mM TCEP, heated to 50 °C before deposition to ensure complete dissolution. To prepare the protein and filling molecule for deposition on gold and ensure that all disulphides were broken, TCEP reducing agent was added to the protein and the filling molecules at a final concentration of 1 mM and incubated at room temperature for 10 min prior to deposition.

A two channel BIAcore X instrument was used for experiments. The protein was injected in a typical volume of 50 μL at a flow rate of 5 μL min^−1^ followed by injections of 1% (w/v) SDS to remove non-specifically bound protein before the filling molecule, thioPEG, was deposited. Three times 50 μL of 0.25 mg mL^−1^ thioPEG in a solution of 1% OG at a rate of 5 μL minute^−1^ was used for each deposition, with a wash of 1% (w/v) SDS between each deposition to remove non-specifically bound thioPEG. Antibodies (IgG) and antigen (human serum albumin, HSA) were diluted in PBS and injected into the flow cell at a rate of 2 μL min^−1^. The running buffer was phosphate buffered saline (PBS, 20 mM sodium phosphate pH 7.6, 137 mM NaCl and 2.7 mM KCl) and the regeneration buffer was 100 mM glycine pH 2.0.

## 4. Conclusions

A scaffold protein with immunoglobulin binding domains separated by a long flexible linker as the functional motif has been successfully produced and shown to form stable self-assembled monolayers on gold. Whilst the dissociation rate of mouse monoclonal IgG from the ZZlinkZZctOmpA is similar to ZZctOmpA, the introduction of a flexible linker between the Z domains increases the capacity for IgG binding resulting in an increase in signal and sensitivity from the array.
